# Successful surgical treatment of impending paradoxical embolism with pulmonary embolism and myocardial infarction

**DOI:** 10.1186/s13019-024-02606-0

**Published:** 2024-03-19

**Authors:** Yong Liu, Zhiyun Yang, Xinxin Sun, Mei Yang, Tao Zhang, Ruilin Li, Ying Wei, Hao Cao

**Affiliations:** 1 Department of Cardiovascular Surgery, Shanghai East Hospital, Tongji University School of Medicine, Shanghai, 200120 China; 2 Department of ICU, Shanghai East Hospital, Tongji University School of Medicine, Shanghai, 200120 China; 3 Department of Cardiology, Shanghai East Hospital, Tongji University School of Medicine, Shanghai, 200120 China; 4 Department of Ultrasound in Medicine, Shanghai East Hospital, Tongji University School of Medicine, Shanghai, 200120 China; 5 Shanghai Heart Failure Institute, Shanghai East Hospital, Tongji University School of Medicine, Shanghai, 200120 China

**Keywords:** Impending paradoxical embolism, Patent foramen ovale, Pulmonary embolism, Myocardial infarction, Cardiac surgery

## Abstract

**Background:**

Paradoxical embolism is a rare cause of acute arterial occlusion. This phenomenon arises when embolic material travels from the venous system crosses an abnormal shunt such as patent foramen ovale, atrial septal defects, ventricular septal defects, or pulmonary arteriovenous malformations, into the arterial system. Impending paradoxical embolism refers to the presence of an entrapped thrombus in the patent foramen ovale.

**Case presentation:**

We report a case of a 68-year-old female patient who presented with an impending paradoxical embolism, alongside both concomitant pulmonary embolism and myocardial infarction with ST-segment elevation. Swiftly addressed through emergency cardiac surgery and systemic anticoagulation, the patient’s condition was effectively treated.

**Conclusions:**

While the ideal treatment strategy for impending paradoxical embolism remains a topic of debate due to limited and inconclusive evidence, emergent open surgery should be contemplated in patients as it signifies a critical clinical emergency.

## Background

Paradoxical embolism (PDE) occurring via a patent foramen ovale (PFO) represents one of the most significant risk factors for systemic embolic occurrences, such as cryptogenic stroke. This phenomenon is rare accounting for less than 2% of all arterial embolism [1]. Its consequences span from stroke [2], to mesenteric artery embolism [3], brachial artery embolism, kidney infarction [4], and in certain instances, even myocardial infarction [5]. The embolus typically comprises a blood clot, although it may be air, a tumor, or a particle of fat. On occasion, a thrombus might also become trapped in a PFO, leading to an impending paradoxical embolism (IPDE) and posing a risk of peripheral arterial embolism. Although rare, this syndrome is potentially life-threatening yet treatable. In reported IPDE cases, clinical presentation varied: about half the patients had a history of isolated pulmonary embolism (PE), in almost 35–40% of cases presented with a combination of PE and PDE, and the remaining cases solely manifested as PDE [6]. Presenting herein is a case of IPDE with pulmonary embolism and myocardial infarction in a 68-year-old female experiencing dizziness and chest tightness. The condition was successfully managed through emergent cardiac surgery and systemic thrombolysis.

## Case presentation

**Fig. 1:**
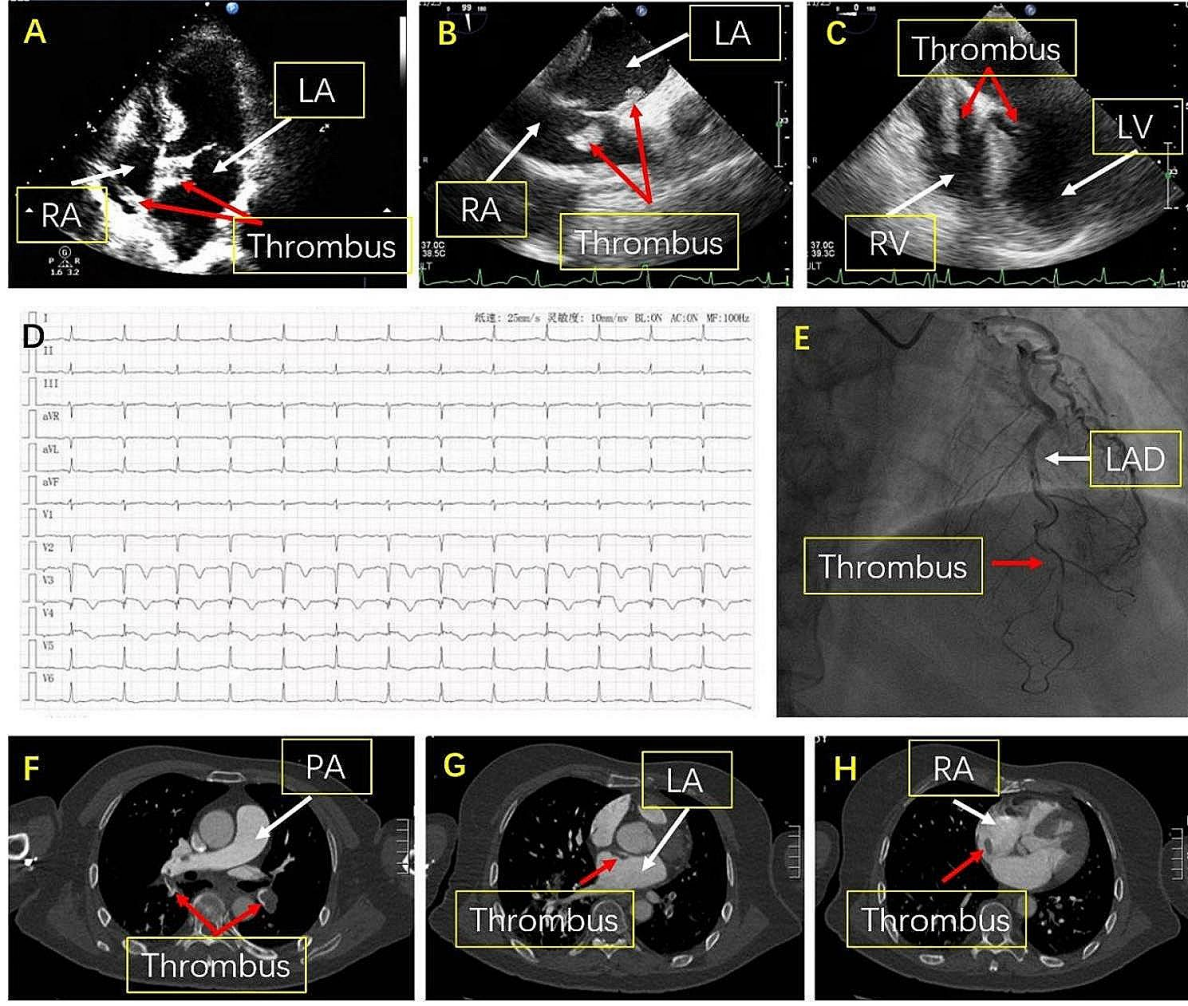
**A**. Transthoracic echocardiography: the thrombus straddling a PFO extending into the RA and LA; the red arrow is pointing at the thrombus in the RA and LA. **B**. Transesophageal echocardiography: the red arrow is pointing at the thrombus stuck in a PFO. **C**. Transesophageal echocardiography: the red arrow is pointing at the thrombus stuck in a PFO moving into ventricle in diastole. **D**. Electrocardiogram on admission: SR 79/min, ST-segment elevation in precordial V1–V4 leads and pathological Q wave in precordial V1–V3 leads. **E**. Coronary angiography: massive thrombotic burden in the middle-distal part of the LAD (the red arrow is pointing at the part of the massive thrombus). **F**. Computed tomography image: a massive thrombus in the pulmonary artery; the red arrow is pointing at the thrombus in the LPA and RPA. **G**. Computed tomography image: the thrombus in the LA; the red arrow is pointing at the thrombus in the LA. H. Computed tomography image: the thrombus in the RA; the red arrow is pointing at the thrombus in the RA. **Abbreviations**: PFO, patent foramen ovale; LA, left atrium; RA, right atrium; LV, left ventricle; RV, right ventricle; SR, sinus rhythm; LAD, left anterior descending artery; LPA, left pulmonary artery; RPA, right pulmonary artery

We present the case of a 68-year-old female patient without significant cardiovascular history admitted to the hospital due to dizziness, chest and back pain, and shortness of breath post-activities. 7 days ago, the patient underwent patellar fracture fixation without using heparin in the Dept. of acute trauma surgery of our hospital. Her medical background encompassed 18 years of hypertension, with a maximum blood pressure of 180/90mmHg, along with a 10-year history of diabetes and cerebral infarction, without any other medical or smoking history. Physical examination revealed no heart murmur, lung moist rale, or leg edema. Her blood pressure registered at 111/66 mmHg, pulse rate was 96 beats per minute, with peripheral artery oxygen saturation at 96% in room air. An arterial blood gas analysis uncovered hypoxemia (PaO2: 61.74mmHg). Transthoracic echocardiography depicted a thrombus straddling the PFO (Fig. 1A), further confirmed by esophageal echocardiography (Fig. 1B) revealing the thrombus entering the ventricle during diastole (Fig. 1C). An electrocardiogram displayed sinus rhythm at 79 bpm and acute anterior septal ST-segment elevation (Fig. 1D). Troponin-T levels significantly rose to 6.860 ng/mL. The coronary angiography demonstrated a thrombus in the left anterior descending artery (LAD) and thrombolysis in myocardial infarction 3 (Fig. 1E). Echocardiography unveiled a decrease in the amplitude of motion of the patient’s middle segment of ventricular septum, and anterior wall and apical segment of left ventricular, resulting in a decreased ejection fraction (EF) of 48%. The serum D-dimer level was significantly increased (> 21.060 FEU). Computed tomography angiography (CTA) scan confirmed thrombi in the left & right pulmonary arteries, left atrium, and right atrium without cerebral infraction (Fig. 1F, G, H).

**Fig. 2:**
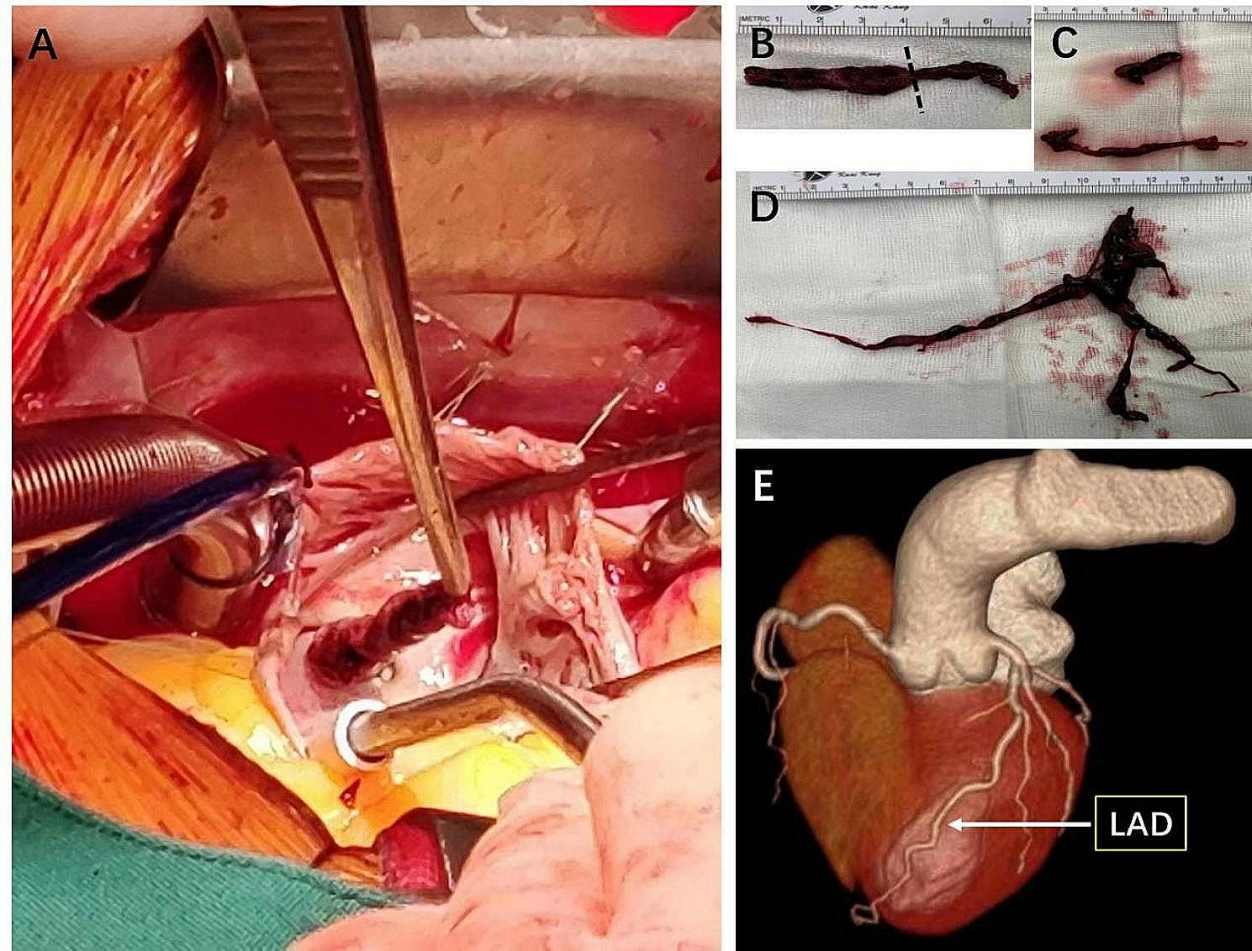
**A**. A very large fresh thrombus in the right atrium, straddling a patent foramen ovale. **B**. The thrombus straddling a patent foramen ovale. (The dashed line indicates the PFO position, The thrombus on the left side of the dashed line is located in the right atrium and the thrombus on the right side of the dashed line is located in the left atrium.) **C**. The thrombus in RPA. **D**. The thrombus in LPA. **E**. Computed tomography Angiography (CTA): the thrombus in LAD disappeared

Thrombolytic therapy wasn’t initially administered due to concerns about potential thrombus fragmentation within the entrapped PFO. The patient underwent emergent cardiac surgery through a median sternotomy with the use of extracorporeal circulation in deep hypothermia (30–32℃) and short total circulatory arrest. Aortic cannulation was typical; 2 venous cannulas were inserted into the inferior and superior vena cava. CO_2_ was used to insufflate the surgical field to reduce the risk of gas embolism. A right atriotomy revealed a notably large fresh thrombus situated within the right atrium, spanning a PFO (Fig. 2A). Extraction of the thrombus followed (Fig. 2B: The dashed line depicts the PFO’s position—the thrombus on the left side of the dashed line in the right atrium and the one on the right side in the left atrium). Upon exploration of the left atrium, no additional thrombus was found, and closure of the atrial septum, including the PFO, was accomplished via direct suturing. Subsequently, upon opening the main pulmonary artery, a substantial volume of coarse thrombus was aspirated from both the left and right pulmonary arteries (Fig. 2C, D). As no thrombus was detected in her lower limb veins via Doppler-ultrasonography of the lower extremities, an inferior vena cava filter was deemed unnecessary.

The patient received therapeutic doses of low molecular weight heparin for three days starting on the first day after the operation. In addition, Rivaroxaban (15 mg, twice daily) was prescribed to maintain anticoagulant therapy. On day 7 post-operation, CTA scan revealed the disappearance of the thrombus in the LAD (Fig. 2E) with a small residual thrombus in the pulmonary artery. Meanwhile, an apex ventricular aneurysm (3 cm×2 cm), a small apical mural thrombus, and an improving EF (54%) were detected by transthoracic echocardiography.

Continuing anticoagulant treatment for another six days, the echocardiography showed the disappearance of the apical mural thrombus, and the patient was subsequently discharged.

## Discussion and conclusions

Paradoxical embolism refers to the clinical phenomenon of thromboembolism that emerges from the venous vasculature, traversing through an intracardiac or pulmonary shunt into the systemic circulation [7]. Initially described by Cohnheim in 1877, diagnosing PDE has remained presumptive due to the difficulty in definitively confirming PDE via a PFO. The diagnosis of PDE depends on the evidence of venous thrombosis or pulmonary embolism, the presence of an intracardiac shunt or a pulmonary fistula, and the presence of arterial embolism. The inaugural case of IPDE was identified by Nellessen et al. in 1985 [8]. IPDE arises when a thrombus-in-transit is stuck into an intracardiac defect. Thrombus-in-transit is a rare but serious manifestation of venous thromboembolism where the thrombus is temporarily lodged in the right heart before entering the pulmonary vasculature. The patient underwent a patellar fracture 7 days ago, and although no pre-operation thrombus was detected via in her lower limb veins by Doppler-ultrasonography in lower extremities pre-operation, lower-limb fractures notably provoke venous thromboembolism, which is clinically manifesting as deep vein thrombosis or PE [9]. The mechanism of IPDE formation in this patient is likely provoked by a thrombus-in-transit from the deep vein of the lower limb falling off and blocking the pulmonary artery partially. This obstruction escalates pressures in the pulmonary arteries, right ventricle, and right atrium, triggering a reversal of the blood shunt via a PFO. A portion of the thrombus-in-transit crossed the PFO from the right atrium to the left atrium but became trapped due to its size. The thrombus-in-transit entering into the left atrium partially falls off and enters the LAD, leading to myocardial infarction.

Three types of thrombus-in-transit observed in the right heart have been outlined using the transthoracic echocardiography: Type A, the most common, are large, freely moving masses highly prone to distal embolization; Type B are small, immobile clots within the right chambers attached to the walls originated in situ; finally, Type C rare and highly mobile, mimick atrial myxomas [10]. These thrombus-in-transit are typically detected via transthoracic echocardiography in patients diagnosed with or suspected of acute PE under clinical conditions. If transthoracic echocardiography fails to identify the thrombi, a transesophageal echocardiography becomes necessary, useful for detecting clots in the pulmonary artery and coexisting PFO.

Multiple treatment options have been described, including anticoagulation alone, systemic thrombolysis, surgical embolectomy, and endovascular catheter-based therapies. Anticoagulation may be universally used unless contraindicated, but it poses risks such as bleeding complications, increased distal embolization, and higher mortality if used alone in more critical patients. Systemic thrombolysis, while it can be administered rapidly at the bedside, especially in a decompensating patient at most/all centers with no special equipment/expertise needed and a lower mortality rate with systemic thrombolysis compared to anticoagulation alone, has limitations as absolute and relative contraindications to thrombolysis exist, bleeding complications, potential ineffectiveness for chronic clots, and distal embolization. Surgical embolectomy is a more definitive therapy, and can be used in the patients if thrombolysis is contraindicated or ineffective and the patients with thrombus-in-transit trapped in PFO, but is more invasive and often limited to tertiary/quaternary centers. Endovascular catheter-based therapies can be used for thrombus-in-transit in the right atrium, right ventricle, inferior vena cava, and pulmonary artery. While versatile, its disadvantages include being more invasive as it requires a veno-venous extracorporeal bypass circuit; needs general anesthesia, and perfusionist; needs transesophageal echocardiography or intracardiac echocardiography guidance; not ideal for right ventricle/acute pulmonary embolism; only for right atrium, inferior vena cava clots, bleeding complications as patients need anticoagulation, and limited availability [11].

There’s no clear consensus on the optimal management of thrombus-in-transit due to the absence of randomized controlled trials comparing treatment strategies. The choice among these methods depends on various factors: patient conditions (comorbidities, hemodynamic status), thrombus characteristics (location, acute/chronic nature, presence in PFO), and institutional capabilities.

IPED emerges as a medical emergency linked to a daunting 11.5% risk of death within 24 h of diagnosis [12]. Among the cases, fifty (13.0%) patients with IPED died during their hospital stay, while 82 (21.2%) experienced acute Ischemic strokes, and 18 (4.6%) were diagnosed with acute myocardial infarction upon admission [12]. A systematic review encompassing 386 IPED cases from 1950 to October 30, 2020, revealed that myocardial infarction, not an ischemic stroke, elevates the risk of death by 8-fold [10].

Despite representing a critical clinical scenario, there’s a scarcity of treatment guidelines and evidence. Available treatment options comprise cardiac surgery, thrombolysis, and anticoagulation, either individually or in combination [1, 13]. However, both thrombolysis and anticoagulation entail potential risks, including bleeding, thrombus fragmentation leading to additional embolisms (pulmonary and arterial), and hemodynamic deterioration. For patients with large and mobile intracardiac thrombi, prioritizing cardiac surgery is crucial, as it enables thrombus removal and PFO closure, significantly averting recurrent paradoxical embolisms, especially in those without comorbidities [14].

Thrombolytic and anticoagulation therapy remains a sensible option for individuals with thrombus adhering to the interatrial septum or faceing high surgery-risk [1]. Although initial studies on aspiration thrombectomy in myocardial infarction with ST-segment elevation demonstrated an improvement in myocardial blush grades and ST-segment resolution rates, larger studies haven’t demonstrated cardiovascular outcomes improved with thrombus aspiration [15]. In this case, only coronary angiography was performed in this patient, and rescue percutaneous aspiration thrombectomy of the coronary artery wasn’t considered due to its limited effectiveness in the treatment of thrombus occlusion during this clinical emergency.

Post-operation transthoracic echocardiography indicated a small apex ventricular aneurysm, apical mural thrombus, and an improving EF recovery. Subsequent anticoagulation therapy led to the disappearance of the thrombus in the LAD and apical mural thrombus.

Our report highlights the successful management of IPDE, coupled with concurrent PE and myocardial Infraction, through cardiac surgery and anticoagulation. Surgical embolectomy proves highly effective in extracting thrombi trapped within intracardiac shunts while directly closing these shunts. Despite ongoing debate over the optimal treatment, a tailored approach combining the merits of both surgical and medical interventions stands to benefit IPDE patients significantly.

## Abbreviations

IPDE: Impending paradoxical embolism
PDE: Paradoxical embolism
PE: Pulmonary embolism
LAD: Left anterior descending artery
EF: Ejection fraction
PFO: Patent foramen ovale
CTA: Computed tomography angiography
